# From study plans to capacity building: a journey towards health equity in cancer survivorship

**DOI:** 10.1007/s10552-023-01808-6

**Published:** 2023-10-18

**Authors:** Prajakta Adsul, Jessica D. Austin, Perla Chebli, Emanuelle M. Dias, Rachel Hirschey, Priyanka Ravi, Aaron T. Seaman, Rosi Vogel

**Affiliations:** 1 Department of Internal Medicine, University of New Mexico, Albuquerque, NM USA; 2 Department of Quantitative Health Sciences, Division of Epidemiology, Mayo Clinic Arizona, Phoenix, AZ USA; 3 Department of Population Health, Grossman School of Medicine, NYU Langone, New York, NY USA; 4 Department of Health Promotion and Behavioral Sciences, Center for Health Promotion and Prevention Research, University of Texas Health Science Center at Houston School of Public Health, Houston, TX USA; 5 School of Nursing, University of North Carolina at Chapel Hill, Chapel Hill, NC USA; 6 Department of Health Promotion Sciences, Mel and Enid Zuckerman College of Public Health, University of Arizona, Tucson, AZ USA; 7 Department of Internal Medicine, Carver College of Medicine, University of Iowa, 200 Hawkins Drive, Iowa City, IA 52242 USA

**Keywords:** Health equity, Community engagement, Survivorship, Reflexivity

## Abstract

This article highlights the importance of pausing and reflecting on one's motivation, capacity, and positionality when engaging in health equity research and encourages researchers to engage in critical self-reflection and contribute to the ongoing dialogue on the ethical conduct of health equity-focused cancer research. In response to the urgent need to address health disparities and improve health equity in cancer survivorship care, the Cancer Prevention and Control Research Network (CPCRN) Survivorship workgroup discussed developing a study focused on understanding how racism impacts patient engagement in cancer survivorship care. However, during the study's development, the workgroup recognized limitations in research team composition and infrastructure. The workgroup engaged in critical self-reflections, individually and collectively, leading to the halting of the research study. Consequently, they redirected their efforts towards strengthening the necessary infrastructure for conducting such research, including diverse investigator representation and equitable partnerships with cancer survivors. The description of this process, along with suggestions for reflection, may be helpful and informative to other researchers and research networks seeking to center marginalized voices and work in partnership to address healthcare and health equity.

**Supplementary Information:**

The online version contains supplementary material available at 10.1007/s10552-023-01808-6.

## Introduction

The disproportionate impact of COVID-19 on communities of color, combined with the murders of Ahmaud Arbery, Breonna Taylor and George Floyd, brought significant attention to health disparities faced by racially and ethnically minoritized populations in the United States [1]. The importance of focusing on the root causes of health disparities in health services research is increasingly recognized [2–4], yet it remains challenging for health equity researchers to fully address the complex social and structural inequities that drive disparities [5, 6]. As with other diseases and health-related conditions, it is imperative that cancer-focused health equity research move from describing outcome disparities to testing actionable multi-level interventions and that the process does not reproduce inequities among researchers or in the community.

Researchers and funders [7, 8] responded with calls for rigorous rapid cycle research methods to improve cancer care delivery [9]. Subsequently, publications about health disparities and equity increased from 86 articles in 2020 to 1538 articles in August 2022 [10]. Many of these publications were from previously unengaged investigators who had pivoted into health equity research without first developing the necessary expertise, giving rise to the term “health equity tourism” by Lett and colleagues [5]. Lett et al. also warned that the misguided “urgency” of research systems that reward productivity puts the impact of and transformation for needed health equity research at risk [5]. While rapid cycle research is critical to improving the speed with which interventions can be translated into practice, building community partnerships, aligning research with such priorities, and ultimately, centering the research processes within the community that benefits from such research are all activities that require careful thought and meticulous planning. Thus, researchers with long-standing, robust partnerships are best positioned to lead health equity-focused rapid cycle research. The urgent—and continual—focus on productivity across disciplines within academic health sciences research can make it difficult for researchers to have the time needed to do health equity research effectively. Alongside encouraging previously unengaged investigators to pivot into health equity research without first developing the necessary expertise, this urgency can also pressure researchers to shorten their engagement, research, and dissemination processes.

Researchers need theoretical and methodological expertise to center communities in health equity research, which Lett and colleagues noted is diminishing in the rise of health equity tourism [5]. Eminent scholars in the field of public health have provided guidance, most notably the Public Health Critical Race Praxis model, highlighting the need to approach research with a race conscious orientation [6]. Additionally, literature about community-based participatory research stresses that reflection must occur among researchers while identifying their positionality in relation to the people, communities, or topics they are studying [11–13], which is different from traditional research approaches [14]. To truly address the historical and social systems that together both reinforce each other and foster discrimination (i.e., structural discrimination) [15], and improve health equity, an “all-hands-on-deck approach” is needed in which individual researchers and teams develop deep understandings of power structures and their positionality to the outcomes that they aim to improve, given their respective positionality and power, and learn to work in ways that they can be most effective [16].

In December 2021, members from the Cancer Prevention and Control Research Network (CPCRN) [17] Survivorship workgroup, consisting of researchers from cancer centers nationwide, convened to develop a research study focused on improving health equity in cancer survivorship care. We, as a Cancer Survivorship Health Equity subgroup, aimed to develop research focused on addressing how racism impacts patient engagement in cancer survivorship care. However, as we discussed potential study ideas, we recognized our limitations related to research team composition and infrastructure. Consequently, we decided to suspend the study's development and redirect our efforts toward improving the infrastructure needed for our team to conduct such research.

In this brief report, we share our critical self-reflections both individually and collectively, as a team of investigators and affiliates representing multiple CPRCN sites, that led us to halt plans for a research study to first focus on ensuring foundational racial and health equity principles were in place for our network [18]. In doing so, we believe that we have further operationalized the race-conscious orientation proposed by Ford and Airhihenbuwa [6]. Our goals in this report are twofold. One, echoing long-standing health equity and community-engaged scholars, we want to re-assert that the work of health equity requires careful labor and that the effort must start in one’s own space. Two, we want to help normalize pausing—and sometimes stopping—research to attend to that careful groundwork. We share how we stopped working on a research study plan and instead re-directed our efforts to increase capacity within our network to do such research. In doing so, we look to contribute to a longer conversation about the ethical conduct of health equity-focused cancer research and resisting the incentivization of continually increased, accelerated research productivity.

## Cancer survivorship health equity group development

Early meetings of the Cancer Survivorship Health Equity subgroup consisted primarily of discussing a shared goal to understand how racism impacts cancer survivorship care. We decided to focus on the experiences of Black and Hispanic/Latino/Latina breast and colorectal cancer survivors based on the workgroup members’ research experiences and community-based networks. We began with brainstorming research questions, guiding frameworks, possible methods, and how to include community partners in our process. We agreed on an overarching study objective: “to describe and assess how individual, interpersonal, community, and societal factors contribute to early-onset cancer survivors’ experiences with and engagement in cancer survivorship care.”

Next, we discussed how to engage community partners who identify as part of and deeply understand the needs and perspectives of our target population (i.e., Black and Hispanic/Latino/Latina breast and colorectal cancer survivors) in determining our research questions, methods, and research conduct. We focused several meetings on whether and how we should form a community advisory board (CAB) of cancer survivors of color. We all had strong partnerships with either individual community members who guide our individual research programs or our affiliated Comprehensive Cancer Centers or Clinical and Translational Science Award programs, which have Community Offices of Engagement with Community Advisory Boards or Patient Advisory Boards. We discussed recruiting CAB members from these existing partnerships. Yet, it was unclear how the research would be conducted across all sites within a large research network, how CAB members would be compensated for their time, and if and how our existing partners from other projects would be included in the CPCRN network. We were concerned about how the CAB members’ time and trust would be protected and respected with expansion from working on local projects with their known research partners to working on a team of national researchers unknown to them. We also considered how our respective CAB members’ community needs and interests in their communities may and may not overlap with national research projects. These concerns prevented us from moving forward with the study design.

We also had several conversations about the lack of racial and ethnic representation across our team and CPCRN investigators at large. We considered our identities, their associated power, and how they were relevant to the work we were considering. Some of the identities we considered across our group include Mexican American, White, cancer caregiver, Arab woman, immigrant, doctoral student, early career faculty, high-risk for breast cancer, and research project coordinator. We reflected on how we have each come to health equity work at different points in our careers for different reasons (see positionality statements in Appendix). Despite a more racially and ethnically representative research team in our initial discussions, our final research team did not include Black researchers. Alongside our concerns about the potential burdens on community members, we felt that moving ahead with the study without the involvement of Black researchers would enact what Petteway refers to as the “dominant ‘ritual’ of public health knowledge production” as from and for a White audience, a reproduction that erases Black researchers and one we did not want to make [19]. This hegemonic form of knowledge production harms Black researchers and the research community, those communities with whom we wish to work, and the public health endeavor, and as a group, we work to avoid its trappings. Our conversations mirrored other ongoing discussions within the CPCRN about improving racial and ethnic representation across the network. We decided we lacked representation within the subgroup needed for the proposed work. Thus, we engaged in conversations with CPCRN leadership and the CPCRN Health Equity workgroup about CPCRN racial and ethnic representation and potential CPCRN CAB formation.

## Applying the CPCRN health and racial equity principles

We collaborated with the CPCRN Health Equity workgroup to apply the newly established CPCRN health and racial equity principles to the processes and activities of our team (See Table 1) [18]. These principles are informed by racial equity theories and frameworks (e.g., Critical Race Theory, Public Health Critical Race praxis, and Intersectionality). The principles are situated in the contemporary understanding of health disparities, which recognize the historic and ongoing racial inequalities in society [8] and provide guidance to help move researchers away from historical approaches that merely document health disparities [7] and instead toward health equity work within an action-oriented paradigm [20]. As described below, we considered how we may apply these principles to revisit our overarching goal of meaningfully conducting research to improve equity in cancer health outcomes across individuals who have been socially assigned to different races.

**Table 1:** CPCRN core health and racial equity principles

1. Engage in power-sharing and capacity building with partners
2. Address community priorities through community engagement and co-creation of research
3. Explore and address the systems and structural root causes of cancer disparities
4. Build a system of accountability between research and community partners
5. Establish transparent relationships with community partners
6. Prioritize the sustainability of research benefits for community partners
7. Center racial equity in cancer prevention and control research
8. Engage in equitable data collection, analysis, interpretation, and dissemination practices
9. Integrate knowledge translation, implementation, and dissemination into research plans

In considering these principles, we identified that we had applied principles three, one, and seven. First, the overarching goal of our project was rooted in principle three (i.e., explore and address the systems and structural root causes of cancer disparities). The overarching objective of our team was to identify how structural influences and experiences in racist systems impact long-term outcomes for cancer survivors. Second, we applied principle one (i.e., engage in power sharing and capacity building) by sharing and discussing our personal positionality, our identities, and the power we see associated with those identities.

Understanding one’s own positionality and power is necessary to engage in power sharing and capacity building. Thus, we each drafted a positionality statement specific to the work we wanted to do together and discussed our reflections to develop a shared understanding of our group identity, enabling us to collectively share power and capacity with future research and community partners. Finally, we applied principle seven (i.e., center racial equity in cancer prevention and control research) through our recognition that the study we had in mind needed to be—indeed, would be harmful if not—conducted collaboratively with culturally, racially and ethnically, and linguistically aligned researchers and cancer survivors. This review solidified our decision not to design a research study focused on our team's understanding of how racism impacts patient engagement in cancer survivorship and to put our efforts into network activities instead focused on improved racial and ethnic representation across the network and development of a network CAB. As a result of this process, three key actions were mobilized in CPCRN: (1) shifting our focus to increase capacity for health equity research; (2) increasing racial and ethnic representation in the network; and (3) developing a network-wide community advisory board. We believe that these three key actions can enhance CPCRN’s impact more broadly and strengthen our collective ability to address cancer survivorship disparities specifically.

### Shifting our focus to increase capacity for health equity research

Considering the recent publication of the CPCRN health equity principles, we decided to not ‘push’ our research objectives forward, but to pause and reflect on our own motivation, capacity, and positionality towards this work. In the process, our group also reflected on the challenges and opportunities of designing and conducting health equity research within a multi-site national network like the CPCRN. Through collaboration with CPCRN’s Health Equity workgroup, we have refocused our efforts to support network activities around increased racial and ethnic representation in the network and development of a structure for a network-wide community advisory board.

### Increasing racial and ethnic representation in the network

Our group was not alone in our recognition of a need for improved racial and ethnic representation among our network and several initiatives were underway to support this need. First, the CPCRN Scholars workgroup was developed, in part, to build the capacity of a diverse cancer research pathway of investigators and practitioners to engage in applied dissemination and implementation work in academic and clinical settings. During the program, scholars are involved in curriculum and webinars to enhance their knowledge in cancer prevention and control and health equity—while also working with CPCRN-affiliated mentors and workgroups (including this one) to increase their connectedness with the network. Our authorship team includes two scholars (ED, PC) from this initiative who will continue to help develop this program by evaluating outcomes from the first three cohorts of scholars and making recommendations for the next cycles of mentoring and curriculum [21, 22]. We recognize that these steps, while important, are just the beginning of the work to shift the power dynamics of public health research.

Second, CPCRN has expanded the affiliate member program, creating a mechanism for researchers who are not employed by one of the eight CDC-funded CPCRN collaborating centers to join CPCRN. This program is critically important to engage diverse expertise across the country, which is also reflected in two of our authorship team members, who are affiliate members with expertise in health equity and implementation science (JA, PA).

### Developing a network-wide community advisory board

In our early meetings we refrained from developing strong research questions and potential methods because we wanted to incorporate the perspectives of Black and Hispanic/Latino/Latina cancer survivor partners, as many of us do in our individual research programs. Practical questions quickly arose around who to invite; how to invite, compensate, and convene; how to avoid asking too much of our existing, already busy partners; and what the CPCRN team of investigators could provide by way of capacity building and service to various members of geographically diverse communities. Through collaboration with CPCRN leadership, we learned that these conversations were also happening across the broader network. Thus, development of a Network-wide CAB was recently discussed at our annual National Network meeting, and several of us have committed to seeing this effort move forward.

## Discussion

A benefit of networks such as the CPCRN is that they can offer the time, space, and resources to facilitate meaningful pauses. Their demands, their goals—and the timelines for achieving those goals—can differ from those of the institutions that compose them. Multi-site national networks are well positioned to increase the capacity to address health inequities by facilitating an environment of cross-fertilization and collaboration of researchers from different institutions with similar research interests. As seen in our experience, CPCRN fostered an environment and protected space for researchers with varying degrees of expertise, to learn and meaningfully engage with each other around their shared interests and goals of engaging in health equity research. It is critical to note the need for structural and institutional changes that would support the capacity to do this work in other settings. For example, institutional change is needed to provide administrative supports and financial payments to non-academic community partners and changes to faculty promotion criteria, considering the time and effort required for quality community-engaged research. This change is especially important for networks whose individual members are hired, housed, and compensated through member institutions.

Still, work within supportive networks is not without challenges, of course. As multi-site national networks like CPCRN continue to push efforts to reduce health inequities across the cancer care continuum, these networks should also reflect on their positionality and capacity to perform this research. As our experience highlighted, and as has been recently recognized by the National Institutes of Health, the contemporary health research workforce lacks diversity in terms of individuals from various lived experiences, thus limiting our ability to propose and conduct research that is critical for addressing the increasing calls to reduce health inequities across the cancer continuum [23]. Moreover, many of these networks consist of researchers or clinicians from funded academic institutions and medical centers. Thus, efforts within and across these multi-site research networks must expand their networks to include patients, advocates, and community partners. To engage in critical reflexivity, researchers may use tools such as the American Medical Association’s Center for Health Justice Community Engagement toolkit (https://www.aamchealthjustice.org/resources/community-engagement-toolkits), the Health Equity Research Impact Assessment [24], and the CPCRN health equity toolkit [18, 25].

Pausing to slow down was our process as a group of researchers from different cancer centers across the country coming together for the first time to engage in health equity research across multiple sites. We worked from our individual positionality to intentionally move forward and gained insights along the way that may benefit other researchers (see Table 2). We also suggest the CPCRN health equity toolkit as a resource with examples and tools that researchers can use [26]. We see the need to pause as particularly important for researchers coming to health equity research for the first time. The pause offers a space to be reflexive about one’s own goals, motivation, and commitment to the work. It may be an opportunity for those who desire to do the work of health equity to avoid the traps of tourism and engage with communities with whom they want to partner. In our case, these processes led us *not* to pursue the research that we originally intended.

**Table 2:** Suggestions to researchers embarking on a new health equity-focused research project

Reflect on your own positionality—lived experience and identities and how you do (or do not) relate to the individuals and communities with whom you want to work
Identify the power associated with your positionality and identify how you may use that power to promote equity initiatives, and engage in power sharing and re-distribution
Consider the collective positionality of your research team and identify if there is a need to develop partnerships with individuals who hold different identities and live experiences
Use health equity-informed principles or theoretical frameworks to guide all phases of research as appropriate, from conception to dissemination

It is plausible that we could have moved forward to explore the experiences of Black and Hispanic/Latino/Latina cancer survivors. However, without this research being conducted in collaboration with Black and Hispanic/Latino/Latina cancer survivors, we believe this could have been yet another example of health equity tourism [6] and that findings may not have been relevant or effective to meaningfully produce needed change. Moreover, proceeding with that work could have harmed researchers and community members in its reproduction of the status quo. Instead, we have identified a critical need for the network to improve the representation of investigators and community partners positioned to examine and mitigate the effects of health inequities and racism in cancer prevention and control. By describing our collective process of reflection, we hope to inspire a broader discussion from researchers in similar positions and engage in a critical reflective dialogue about health equity research. As our working group and the CPCRN shift our focus to meet these needs, our work’s impact is far more effective in the long term as we can lay the foundation for a multitude of future research projects.

### Supplementary Information

Below is the link to the electronic supplementary material.

Supplementary file1 (DOCX 24 KB)

## Data Availability

Data sharing not applicable to this article as no datasets were generated or analyzed during the current study.
