# Supplementary material for: From study plans to capacity building: a journey towards health equity in cancer survivorship
Source: Cancer Causes Control. 2023 Oct 18;34(Suppl 1):7–13. doi: 10.1007/s10552-023-01808-6 (PMC10689513; doi:10.1007/s10552-023-01808-6)
Supplement: Supplementary file 1 — Supplementary file1 (DOCX 24 KB) [file 10552_2023_1808_MOESM1_ESM.docx]

**Supplementary Material**

Positionality Statements

Prajakta Adsul: I am a cisgender, heterosexual woman. I spent my childhood and young adulthood as a *Dalit* in India, which influenced my understanding of society as well as my practice as a medical doctor. Today I am a recent citizen of the United States and a faculty at a research-intensive institution. Much of my research portfolio is geared towards engaging partnerships in understanding disparities and co-developing interventions to promote health equity, with a specific focus on implementation science. For me, implementation science provides a strategy to achieve equity, only and only if the focus is on addressing systemic barriers for underserved populations and resource-limited settings. These research methods and theories allow me to ground my scientific curiosity in the pursuit of equity and humbles me in recognizing that there is a lot to learn from people with lived experiences, partners delivering healthcare, and researchers from across the health and non-health focused disciplines.

Jessica Austin: I am a White, cis-gender, heterosexual woman, cancer caregiver, who is high risk for breast cancer, and an early career Assistant Professor working for a nationally recognized health care system in the U.S. I am the first in my family to get an advanced degree and the first woman to get a college degree. Growing up in the South, I experienced the power of implicit biases and gender norms. At once low-income, uninsured, and now, a woman of child-bearing age, I’ve experienced challenges accessing health care. These experiences fuel my passion towards health equity research and the belief that access to care is a right and not a privilege. My research uses implementation science principles to optimize the delivery of cancer care innovations in under-resourced, historically underserved populations. Implementation science provides me a lens for understanding the importance of context in developing equitable strategies to improve care. Yet, my research requires building trusting relationships with populations that don’t look like me or speak like me. Trust building takes time, and this process is frequently out of touch with the timelines of funding structures and the pressure of producing as an early-career investigator. Because there I hold unearned power as a White, heterosexual, cis-woman, I struggle with my positionality as an outsider and constantly wonder if I am ‘allowed’ to or am the ‘right’ person to do this work. I came to this group eager to reflect on these curiosities with others engaged in this work.

Perla Chebli: I engage in heath equity work as an Arab woman, an immigrant, the daughter of middle-class educators, a citizen of what they call a “third world country”. Yet, I also had the opportunity to pursue higher education in the United States, earn advanced degrees, and land an Assistant Professor position at a research-intensive institution in New York City. I recognize that the duality of my experiences confers upon me both shared struggles with historically minoritized communities and unique privileges. I allow this tension to guide my work and thinking around health equity, so I may constantly challenge myself to reflect on how my identities and experiences may be shaping the way I see the world. I also recognize that positivist science perpetuates a hierarchy of knowledge slanted towards researchers; therefore, I am committed to authentic partner engagement to center community voices, uncover fundamental determinants of cancer disparities, and identify and implement responsive strategies. My involvement in the CPCRN Cancer Survivorship workgroup is peripheral – as a member of the Health Equity workgroup, I participated in the development of health equity principles for the CPCRN. My role is to help contextualize how these principles can be leveraged to design an equity-focused study protocol.

Emanuelle Dias: As I reflect on my interest in health equity research, I think about coming home from school one afternoon as a nine-year-old and learning that my family and I would move to Brazil for six months so my uninsured mother could receive breast cancer treatment and care. It wasn’t until I was a junior in college taking an introduction to health disparities course that I connected that defining moment in my life with my career goals. Today, as a predoctoral fellow focused on health equity, implementation science, cancer prevention, and research conducted in resource-constraint settings, I joined this workgroup as a Cancer Prevention and Control Research Network (CPCRN) scholar to learn more about health equity specifically in the context of cancer survivorship and patients of color. I also bring the identities of being a young scientist, anti-racist ally, and first-generation American and student. These identities intersect with my privileges of being a light-skinned Latina and a heterosexual cis-gender female who was raised in a Christian home. I have learned a tremendous amount from the other members in this group and we have actively discussed the health equity perspective and recognize that health is not only impacted by individual behaviors, but by broader systems and processes that may create barriers to performing those behaviors that impact health. I will strive to continue to create inclusive, diverse, and welcoming spaces with many racial, ethnic, and social identities represented. I am also committed to supporting and mentoring the next generation of students because it is through a diverse workforce that we can create a more equitable future and meet the ever-changing health needs of various communities across the United States.

Rachel Hirschey: I continuously work to understand where I sit in the system of racism and how I can use the power associated with my various identities to structure my research, teaching, service, and citizenship to deconstruct racism. My research program is focused on achieving cancer health equity across patients who are socially assigned to different races. As a health equity researcher, I joined this workgroup because I wanted to work a national team of investigators to focus specifically on understanding how experiences in a racist system impacts engagement in cancer survivorship care for patients of color. I want to use these findings to work on rebuilding cancer care to be inclusive and equitable. The relevant identities that I bring to this group include being White, cis-gender female, heterosexual, non-religious, first-generation – educated, a nurse, a scientist and an anti-racist. As I do anti-racist work of any form, I strive to remain mindful of the limitations that I have as a White individual. Simultaneously, I work to identify the ways in which I also have unique opportunities and responsibilities to contribute to, and lead anti-racist work. I strive to work with teams that are racial, ethnically, and professionally diverse, because I believe that deconstructing racism is the work of all, yet *how* a given individual can be effective in this work is in part based on their social location.

Priyanka Ravi: I am a cis-gender, heterosexual, female, dentist, public health professional, and doctoral student. I grew up in south India, graduated as public health dentist and worked in oral cancer prevention research in different parts of India. The experience of working in different health care setting in different states of India with varied language, cultural, religious, and social backgrounds has helped me focus my research on the social factors impacting health care access among marginalized population. Personally, I lost two loved ones in my family to cancer. My family underwent physical, emotional, and financial problems from the time of diagnosis to treatment, cancer survivorship, caregiving, and making end of life decisions. We identify the biological factors but fail to notice the social factors around the cancer survivorship research. As a doctoral student, I am learning about the systemic health care problems in the United States, working with the CPCRN cancer survivor group has improved my understanding on the health equity among cancer survivors in the United States. With my interest in global health and cancer prevention, I am interested to learn the factors that impact the health system. I plan to work as a global cancer prevention researcher with focus on health equity.

Aaron Seaman: I am a large (6’2”, 225lb), White, cisgender, heterosexual man in his mid-40s. Upon entering a room, I try to be aware of the power dynamics that enter alongside me, as part of me. I have always been uncomfortable with those dynamics; I try not to “man spread,” but rather to take up less space, to track who talks most in a conversation, to listen, to hear. Often, I do so imperfectly, and many times that is not the answer. I am increasingly aware that, to be an ally means also knowing when and in what spaces to marshal my identities, to take up more space in service of resistance and change. Professionally, I am medical anthropologist and health services researcher, with a tenure-track position at a research-intensive academic medical center. I also am a first-generation college student, and 30 years in, I still feel uncertain in academic spaces most of the time. Anthropology gives me a framework and language for thinking about structure and power, how the conditions of possibility for personal or community agency are shaped and constrained by them. It gives me a methodology and tools for engagement and for examining those relationships and the disparities they maintain across scales. However, it also carries along with it a colonial past-into-present that too often leads to extractive research relationships that result in tenure, othering, and little more. Health equity work offers the possibility of another way to envision research relationships, and it is central to doing the work to improve cancer care delivery. I am admittedly still learning, a noob as my child would say, but eager to do so. And I’m committed to mentorship and to building the infrastructure to support a research world that reflects the diversity of the communities we live and work within and alongside.

Rosi Vogel: I am a Mexican American woman who works in community outreach and engagement with mostly Hispanic and Latin communities in Southern Arizona. One of the first things I noticed when I moved to the United States from Mexico was that when filing paperwork, there was a question about race, this question confused me for I didn’t know how to answer. My research work allows me to interact directly with people living in border towns, most of them of Mexican origin, but others from other towns in Latin America. When I was using the data collection instruments designed by cultural adaptation experts, I realized that they were not cultural appropriate and confirmed that among others, the race question was not only confusing to me, but to the majority of the participants; some would answer “I don’t know” others will say “Mexican?”, and others would tell me “you chose for me”. The question of ethnic or cultural background resonated more with them. Some identified as Hispanics, others as Latinos, others as Mexicans. I joined this group because I felt that many researchers want to work with the Hispanic community but do not have the right team, they don’t know the culture, or don’t invest the time to create relationships and earn trust. This exercise takes time. My position is that we can’t address Hispanic communities as a homogenous group, we should foster a bidirectional collaboration and engage them in the design of the research questions that make sense to them. I know that not all people of Mexican origin see themselves as disadvantaged, or poor or uneducated, these characteristics have negative connotations. Instead, they are under-resourced. I know that me being Mexican doesn’t make me an expert because my living experiences are unique to me. I am aware that there is discrimination among Hispanics, Mexicans, and so forth, therefore we not only need a diverse representation in research teams, but we also need to always engage with our community partners that live in the communities that we want to work with. We need to reflect on our mission and goals, and apply health equity and antiracism principles in the most responsible, honest, and meaningful way, even if it takes time to do it right. All these statements reflect my work experience.
